# Supplementary material for: HMMRATAC: a Hidden Markov ModeleR for ATAC-seq
Source: Nucleic Acids Res. 2019 Jun 14;47(16):e91. doi: 10.1093/nar/gkz533 (PMC6895260; doi:10.1093/nar/gkz533)
Supplement: gkz533_Supplemental_File [file gkz533_supplemental_file.pdf]

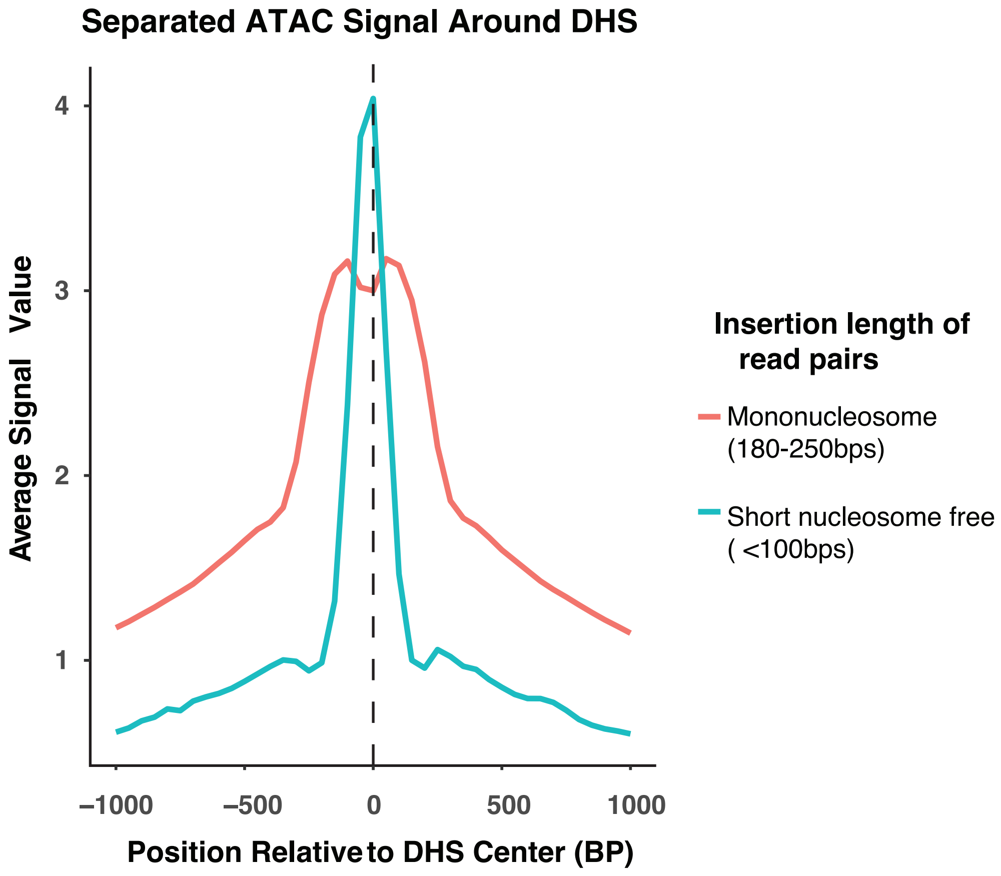

Supplemental Figure S1. Nucleosome-free and mono-nucleosome ATAC-seq fragments around DNaseI Hypersensitive sites.

ATAC-seq signals, separated by fragment length, around DNase hypersensitive sites (DHS). Short nucleosome free reads are from read pairs with insertion length below 100 bps, mononucleosome reads are from those between 180 and 250 bps in length.

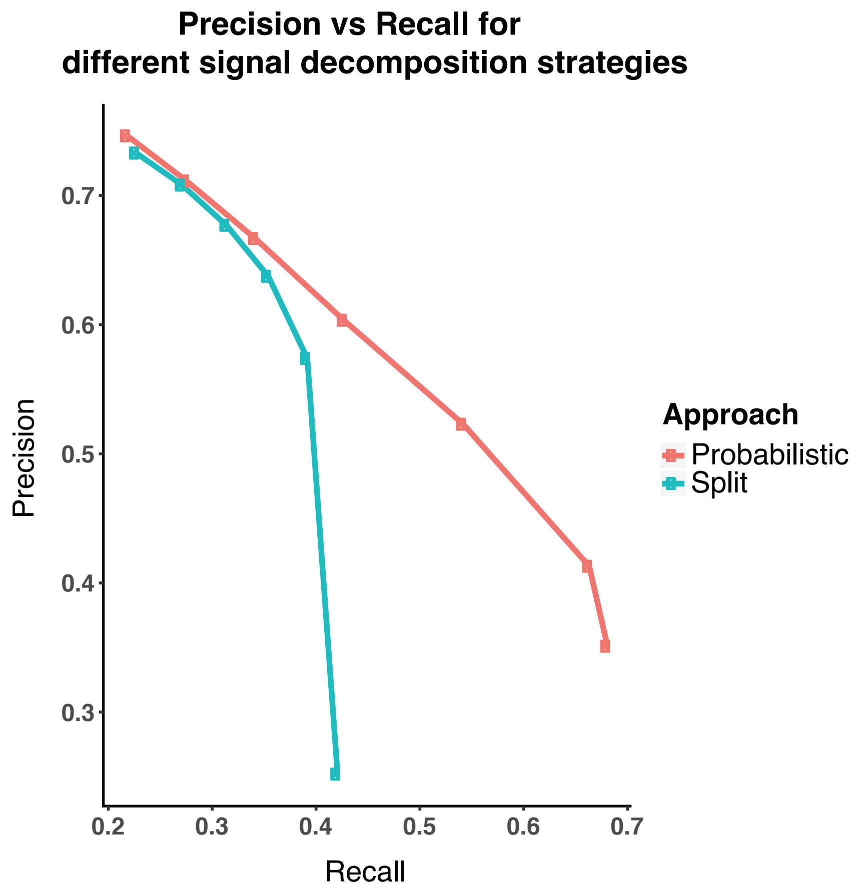

Supplemental Figure S2. HMMRATAC probabilistic approach versus cutoff-based approach.

Precision-Recall plots while using chromatin states as gold standard, comparing the probabilistic approach to signal decomposition used by HMMRATAC versus an alternative strategy where signal decomposition is accomplished using the cutoffs determined by Buenrostro et al.

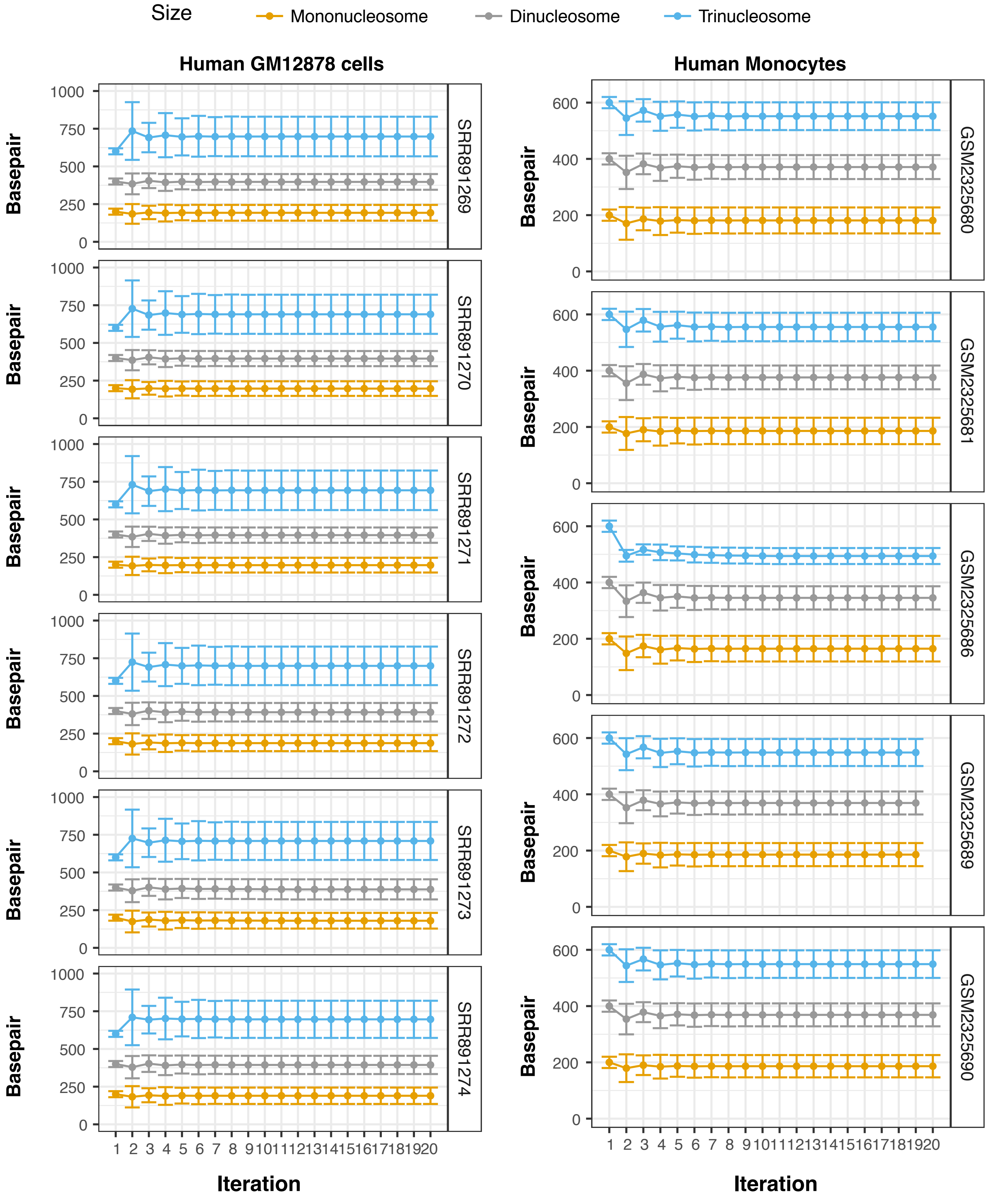

Supplemental Figure S3. Mean and standard deviation parameters per EM iteration for three nucleosomal distributions, on human GM12878 and monocytes ATAC-seq data. Note, the iteration #1 is in fact the initial parameters.

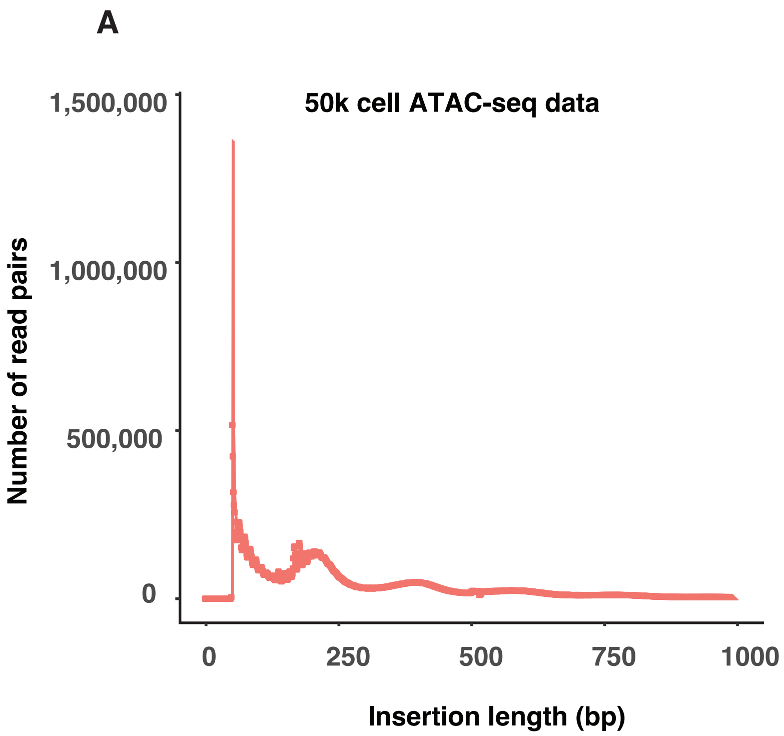

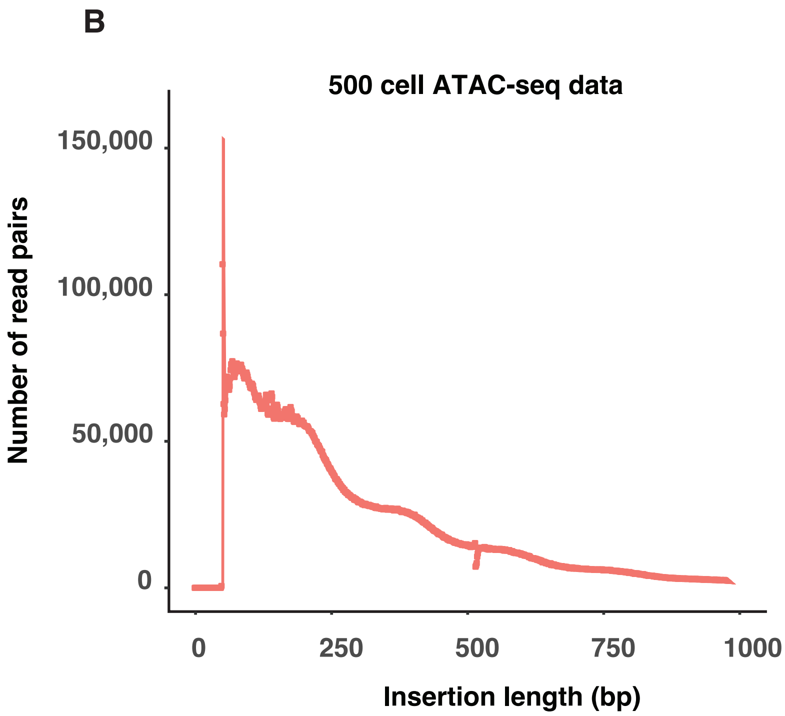

Supplemental Figure S4. Fragment length distribution for 50,000 cells per replicate and 500 cells per replicate.

Fragment length distributions were calculated from ATAC-seq assays conducted by Buenrostro et al. for 50,000 cells per replicate (**A**) and 500 cells per replicate (**B**).

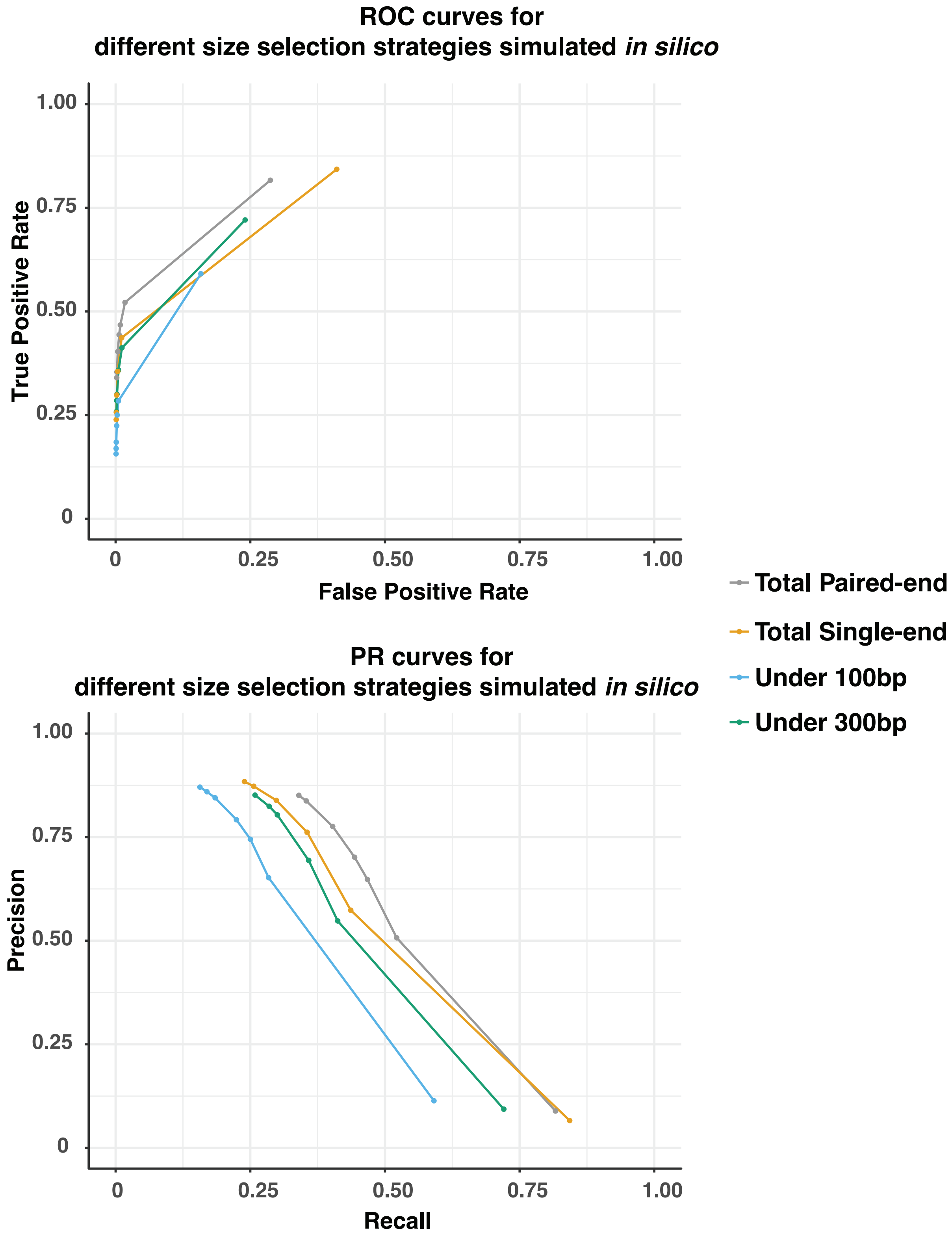

Supplemental Figure S5. Effects under difference size-selection (*in silico simulation*) strategies.

We used chromatin states in GM12878 as gold standard, comparing the different size-selection strategies by simulating Buenrostro et al. data *in silico*. We have four sets of data: 1) total reads with pairing information; 2) total reads without pairing information; 3) only read pairs with insertion size less than 100bp long; 4) only read pairs with insertion size less than 300bp long.

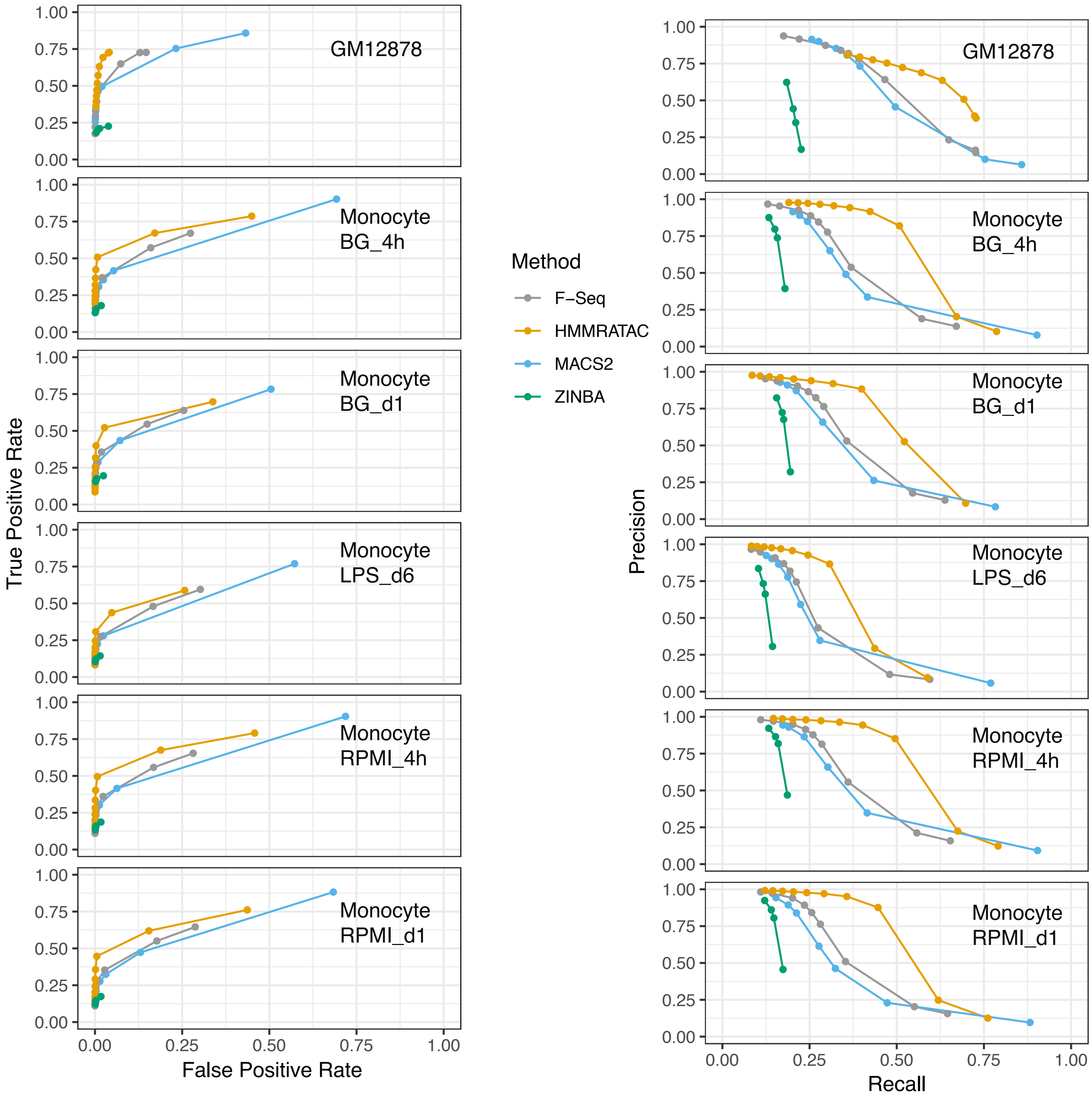

Supplemental Figure S6. ROC curves and PR curves for testing HMMRATAC, F-Seq, MACS2 and ZINBA

Same as the main Figure 4. Left: Receiver Operator Characteristic (ROC) curves and right: precision-recall (PR) curves for the real positive and real negative pairs of 1. active chromatin states vs. heterochromatin for GM12878 cells, 2. active histone marks (either H3K4me1, H3K4me3 or H3K27ac) vs. heterochromatin (H3K9me3) for human monocytes. Consistent with the comparison of peak callers for DNase-seq analysis (43), ZINBA underperforms other methods by a big margin. So we didn't include ZINBA in other analyses in this manuscript.

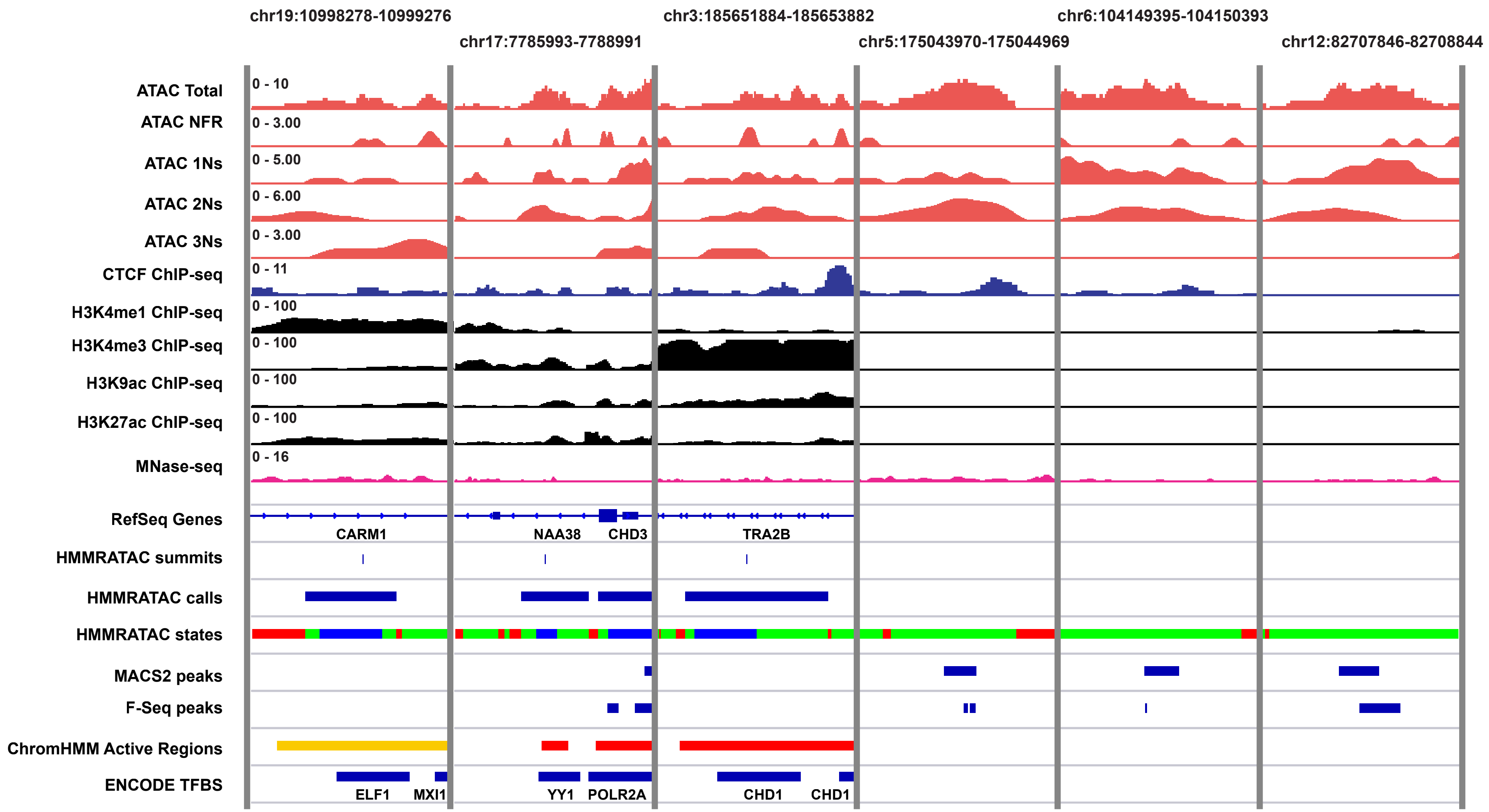

Supplemental Figure S7. Inconsistent calls by MACS2, F-Seq, and HMMRATAC

We showed here six inconsistent calls by MACS2, F-Seq, and HMMRATAC. The left three regions show accessible chromatin called by HMMRATAC but not by conventional peak callers. They not only overlap with active chromatin state and known TFBS (GM12878 cell line) annotated by ENCODE but also show a typical chromatin structure characterized by nucleosomal signals surrounding NFR signals. The right three regions are called by MACS2 and F-Seq but not by HMMRATAC. They can't be supported by chromatin state or TFBS evidence and lack the pattern that HMMRATAC can recognize. Same as Figure 2, in the HMMRATAC states track, "red" represents "background state", "green" represents "nucleosome state", and "blue" represents "center state"; in the ChromHMM Active Regions track, "red" represents "Active Promoter" and "yellow" represents "Strong Enhancer" state.

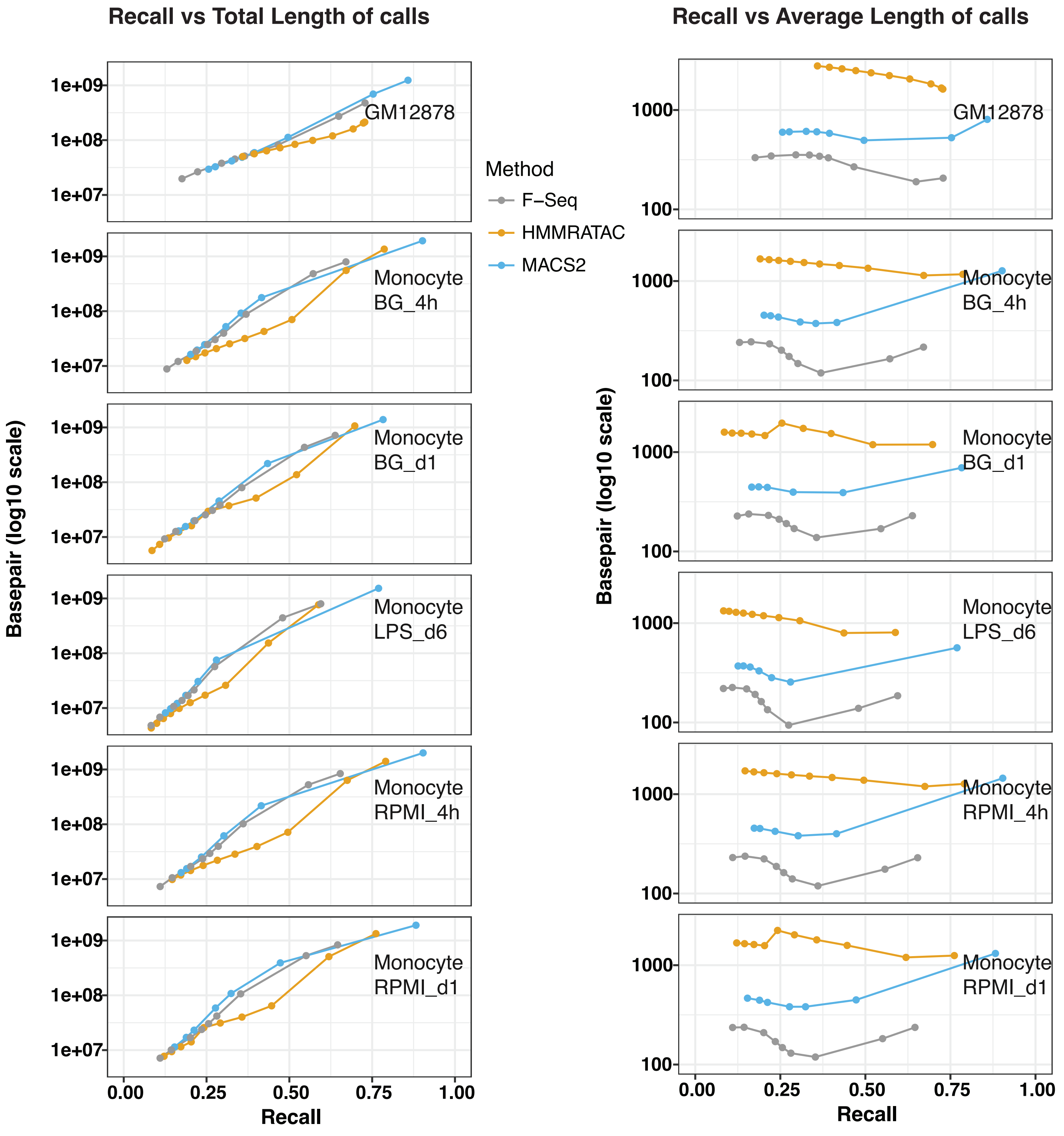

Supplemental Figure S8. Recall/Sensitivity vs total length of calls or average length of calls from HMMRATAC, MACS2 and F-Seq

The testing data, gold standard and real negatives are the same as Figure 4. Y-axis is in log10 scale.

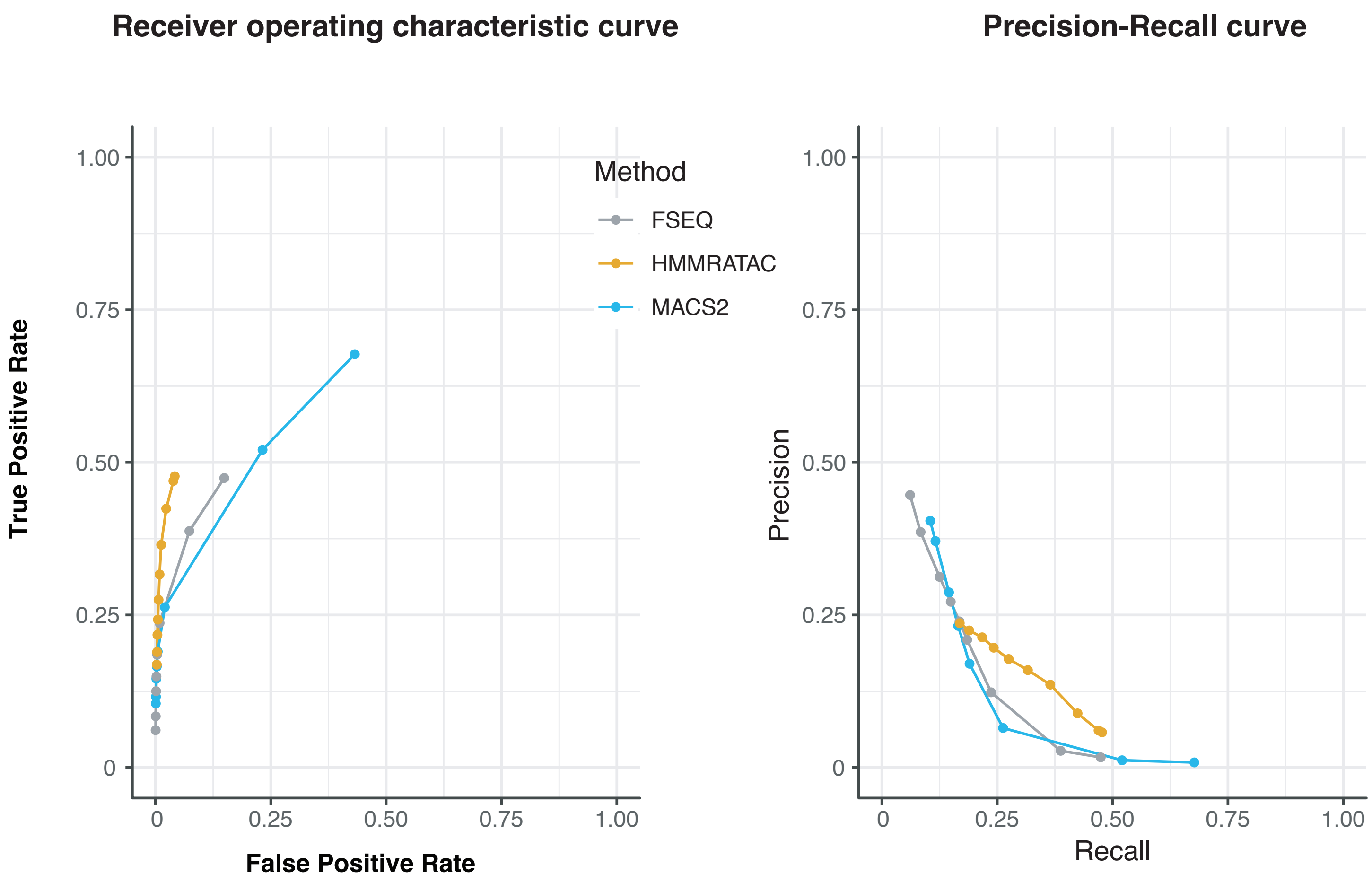

Supplemental Figure S9. ROC curves and PR curves when testing super enhancers

Evaluate methods on ATAC-seq data from GM12878 cell line while using super enhancers as true set, and heterochromatin chromatin states as false set.

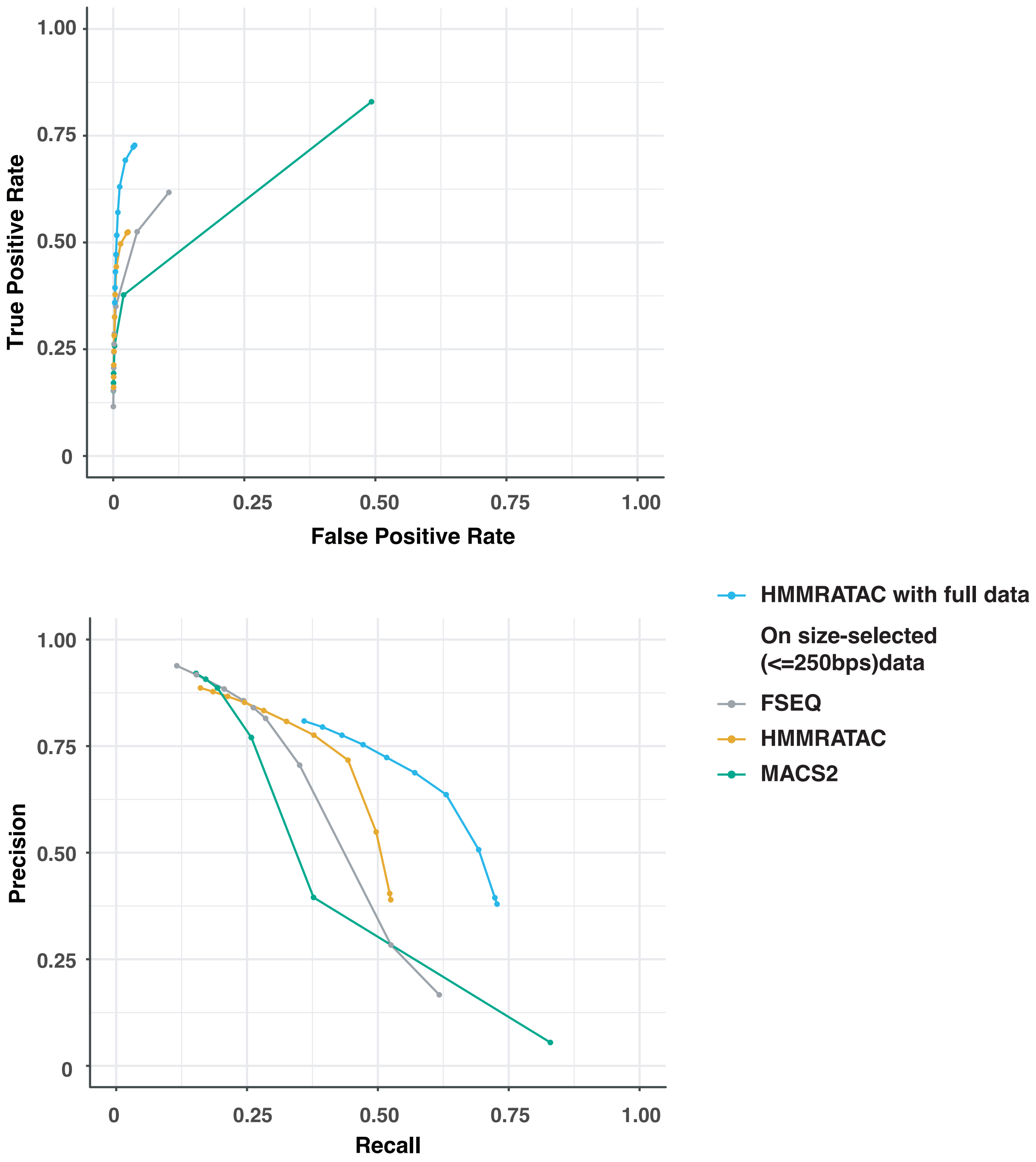

Supplemental Figure S10. ROC curves and PR curves for testing HMMRATAC, F-Seq and MACS2 on size-selected data from GM12878 ATAC-seq

We used chromatin states in GM12878 as gold standard as Figure 4, comparing the performance of different methods on the size-selected (<=250 bps) ATAC-seq data. Although the overall performance decreases compared with full dataset (HMMRATAC with full data vs HMMRATAC), HMMRATAC still outperforms other methods.

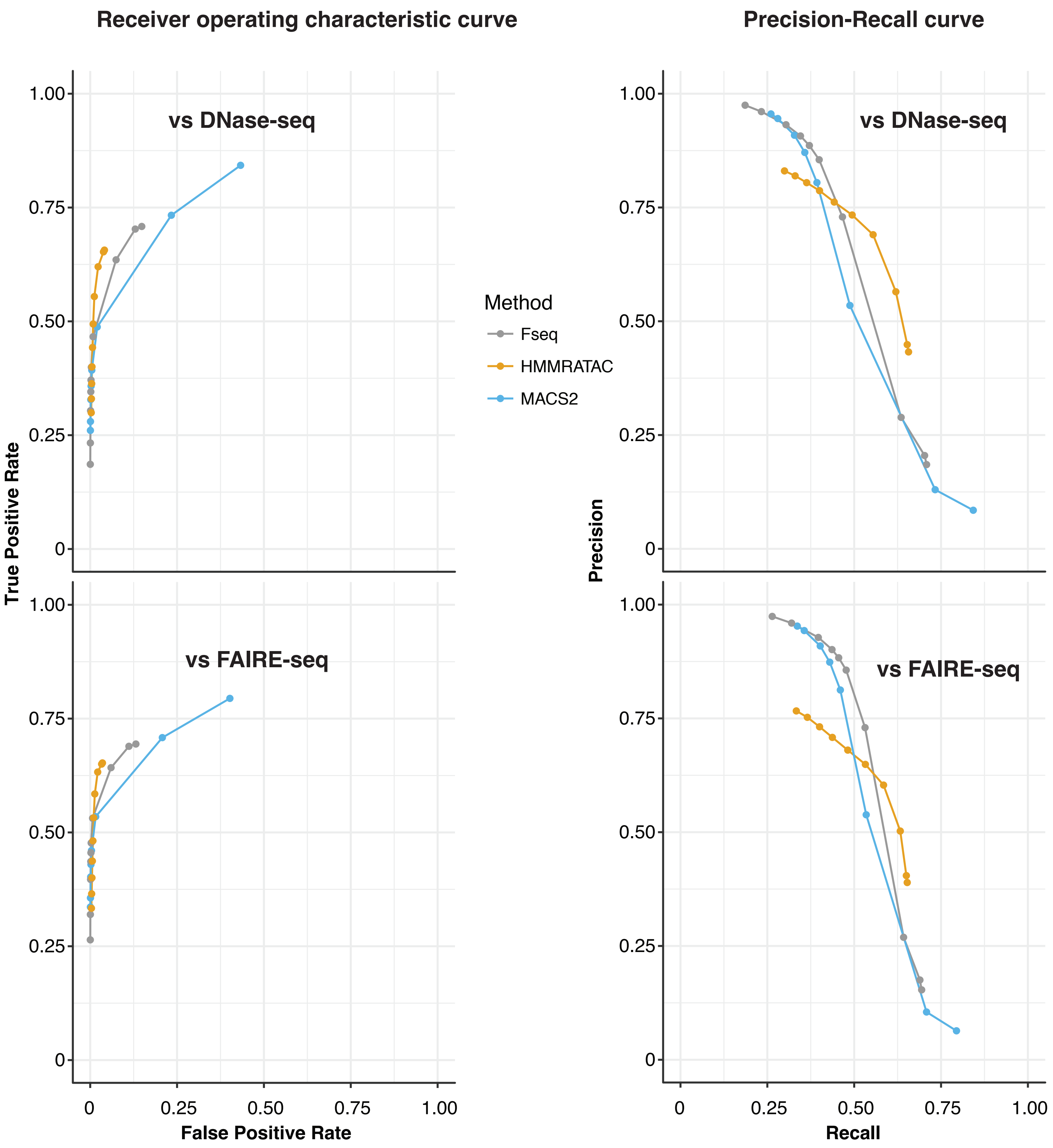

Supplemental Figure S11. ROC curves and PR curves with DNase-seq or FAIRE-seq as standard

Evaluate three methods on ATAC-seq data from GM12878 cell line. This figure shows ROC curvesand PRCs while using DNase-seq or FAIRE-seq peak calls from ENCODE as gold standard. The real negative set was defined as heterochromatin state from chromatin state analysis that do not overlap with any DNase-seq or FAIRE-seq peak calls, respectively.

		Transition parameters			Emission parameters				
run	state	Background	Nucleosome	Center	NFR	1N	2Ns	3Ns	
1	Background	0.333	0.333	0.333	0.332	0.684	0.608	0.794	from K-means
	Nucleosome	0.333	0.333	0.333	0.863	1.731	1.913	2.574	
	Center	0.333	0.333	0.333	2.408	4.148	4.033	4.368	
	Background	0.951	0.049	0.001	0.064	0.26	0.541	0.773	from Baum-Welch
	Nucleosome	0.038	0.958	0.005	0.595	1.153	0.839	1.07	
	Center	0.001	0.009	0.99	1.527	2.75	2.856	3.436	
2	Background	0.333	0.333	0.333	0.332	0.684	0.607	0.794	from K-means
	Nucleosome	0.333	0.333	0.333	0.863	1.73	1.912	2.575	
	Center	0.333	0.333	0.333	2.409	4.148	4.031	4.37	
	Background	0.95	0.049	0.001	0.063	0.257	0.54	0.773	from Baum-Welch
	Nucleosome	0.038	0.958	0.005	0.594	1.152	0.838	1.07	
	Center	0.001	0.009	0.99	1.526	2.748	2.853	3.436	
3	Background	0.333	0.333	0.333	0.332	0.684	0.606	0.795	from K-means
	Nucleosome	0.333	0.333	0.333	0.863	1.73	1.909	2.577	
	Center	0.333	0.333	0.333	2.409	4.148	4.027	4.375	
	Background	0.95	0.049	0.001	0.063	0.257	0.538	0.774	from Baum-Welch
	Nucleosome	0.038	0.958	0.005	0.594	1.152	0.837	1.071	
	Center	0.001	0.009	0.99	1.526	2.748	2.85	3.439	

Supplemental Table S1. Robustness to random initialization. We ran k-means clustering and HMM training three time with random initializations. We found the k-means results and the final HMM parameters are mostly identical among the three runs.

	Transition			Emission				
	**1 background**	**2 nucleosome**	**3 center**	**NFR**	**N1**	**N2**	**N3**	**Sample**
**1 background**	0.941646494	0.057626682	7.27E-04	0.00809468	0.00168701	0.26660351	0.51607081	GM12878_50k
**2 nucleosome**	0.022444289	0.973686683	0.00386903	0.44833745	0.91141612	0.75022441	0.71305135	GM12878_50k
**3 center**	8.97E-04	0.008881959	0.99022132	1.42875405	2.48954314	2.32517186	2.67086374	GM12878_50k
**1 background**	0.973592154	0.013258623	0.01314922	0.26534267	0.53730088	0.1012532	0.01111763	GM12878_500
**2 nucleosome**	0.007559538	0.947031141	0.04540932	0.10623542	0.4278873	0.94871716	0.88893075	GM12878_500
**3 center**	0.006251221	0.038300922	0.95544786	0.733387	1.29281661	1.18487558	1.08560785	GM12878_500
**1 background**	0.94108237	0.049334524	0.00958311	6.44E-154	6.88E-155	6.92E-160	6.15E-165	Monocyte_RPMI_d1
**2 nucleosome**	0.033945785	0.949682566	0.01637165	0.58035196	1.09937253	0.0198803	4.34E-06	Monocyte_RPMI_d1
**3 center**	0.004682234	0.012768725	0.98254904	0.75578354	1.45120846	1.12300594	0.30983384	Monocyte_RPMI_d1
**1 background**	0.942323253	0.050284474	0.00739227	0	0	0	0	Monocyte_RPMI_4h
**2 nucleosome**	0.036632826	0.948028679	0.01533849	0.53024286	1.05692383	0.0203743	3.60E-06	Monocyte_RPMI_4h
**3 center**	0.004923992	0.012448087	0.98262792	0.82656381	1.66069885	1.2126787	0.30101529	Monocyte_RPMI_4h
**1 background**	0.940676194	0.052896057	0.00642775	0	0	0	0	Monocyte_LPS_d6
**2 nucleosome**	0.052891754	0.934903796	0.01220445	0.69024376	0.90409209	0.01901364	9.53E-11	Monocyte_LPS_d6
**3 center**	0.007947584	0.015723289	0.97632913	0.73380067	1.23830927	0.95008077	0.20874454	Monocyte_LPS_d6
**1 background**	0.965517163	0.028609696	0.00587314	0	0	0	0	Monocyte_BG_d1
**2 nucleosome**	0.05141867	0.935152294	0.01342904	0.54936015	0.8994144	0.01724658	7.81E-06	Monocyte_BG_d1
**3 center**	0.008998123	0.010309457	0.98069242	0.6529295	1.19554243	0.99172202	0.32536661	Monocyte_BG_d1
**1 background**	0.935112863	0.0558214	0.00906574	0	0	0	0	Monocyte_BG_4h
**2 nucleosome**	0.043358826	0.940702059	0.01593911	0.64883274	0.98375378	0.01902861	4.94E-06	Monocyte_BG_4h
**3 center**	0.005043926	0.013647117	0.98130896	0.76665595	1.40078975	1.05988359	0.32775993	Monocyte_BG_4h

Supplemental Table S2. HMM parameters from HMMRATAC Baum-Welch training on ATAC-seq data of GM12878 and monocytes. The “NFR” column is for nucleosome free region signals, “1N” is for mono-nucleosome, “2Ns” is for di-nucleosome and “3Ns” is for tri-nucleosome signal.

CistromeID	GEO Series	GEO Sample	Celltype	Tissue	Experiment Type	Condition
65282	GSE87218	GSM2325690	Monocyte	Blood	ATAC-seq	ATAC_RPMI_d1_8451
66431	GSE85246	GSM2262990	Monocyte	Blood	H3K27ac	RPMI_d1_rep2_H3K27ac
66436	GSE85246	GSM2262970	Monocyte	Blood	H3K27ac	RPMI_d1_rep1_H3K27ac
72242	GSE85246	GSM2263025	Monocyte	Blood	H3K4me1	RPMI_d1_rep2_H3K4me1
72245	GSE85246	GSM2263022	Monocyte	Blood	H3K9me3	RPMI_d1_H3K9me3
72249	GSE85246	GSM2263016	Monocyte	Blood	H3K4me3	RPMI_d1_rep1_H3K4me3
72259	GSE85246	GSM2263003	Monocyte	Blood	H3K4me1	RPMI_d1_rep1_H3K4me1
72277	GSE85246	GSM2262978	Monocyte	Blood	H3K27me3	RPMI_d1_H3K27me3
72281	GSE85246	GSM2262973	Monocyte	Blood	H3K4me3	RPMI_d1_rep2_H3K4me3
65283	GSE87218	GSM2325689	Monocyte	Blood	ATAC-seq	ATAC_RPMI_4h_8448
66423	GSE85246	GSM2263019	Monocyte	Blood	H3K27ac	RPMI_4h_rep1_H3K27ac
66435	GSE85246	GSM2262977	Monocyte	Blood	H3K27ac	RPMI_4h_rep2_H3K27ac
72244	GSE85246	GSM2263023	Monocyte	Blood	H3K4me1	RPMI_4h_rep2_H3K4me1
72253	GSE85246	GSM2263012	Monocyte	Blood	H3K9me3	RPMI_4h_H3K9me3
72262	GSE85246	GSM2262998	Monocyte	Blood	H3K4me3	RPMI_4h_rep2_H3K4me3
72269	GSE85246	GSM2262989	Monocyte	Blood	H3K27me3	RPMI_4h_H3K27me3
72278	GSE85246	GSM2262976	Monocyte	Blood	H3K4me3	RPMI_4h_rep1_H3K4me3
72300	GSE85246	GSM2262948	Monocyte	Blood	H3K4me1	RPMI_4h_rep1_H3K4me1
65286	GSE87218	GSM2325686	Monocyte	Blood	ATAC-seq	ATAC_LPS_d6_8455
66415	GSE85246	GSM2263053	Monocyte	Blood	H3K27ac	LPS_d6_rep2_H3K27ac
66440	GSE85246	GSM2262956	Monocyte	Blood	H3K27ac	LPS_d6_rep1_H3K27ac
72228	GSE85246	GSM2263046	Monocyte	Blood	H3K4me3	LPS_d6_rep2_H3K4me3
72232	GSE85246	GSM2263041	Monocyte	Blood	H3K9me3	LPS_d6_H3K9me3
72238	GSE85246	GSM2263031	Monocyte	Blood	H3K4me3	LPS_d6_rep1_H3K4me3
72257	GSE85246	GSM2263005	Monocyte	Blood	H3K27me3	LPS_d6_rep1_H3K27me3
72264	GSE85246	GSM2262996	Monocyte	Blood	H3K4me1	LPS_d6_rep1_H3K4me1
72287	GSE85246	GSM2262964	Monocyte	Blood	H3K27me3	LPS_d6_rep2_H3K27me3
72296	GSE85246	GSM2262953	Monocyte	Blood	H3K4me1	LPS_d6_rep2_H3K4me1

Supplemental Table S3. The accession numbers for histone modification ChIP-seq datasets used to evaluate ATAC-seq analysis in human monocytes.

65291	GSE87218	GSM2325681	Monocyte	Blood	ATAC-seq	ATAC_BG_d1_8453
66432	GSE85246	GSM2262988	Monocyte	Blood	H3K27ac	BG_d1_rep1_H3K27ac
66445	GSE85246	GSM2262937	Monocyte	Blood	H3K27ac	BG_d1_rep2_H3K27ac
72230	GSE85246	GSM2263043	Monocyte	Blood	H3K4me3	BG_d1_rep1_H3K4me3
72233	GSE85246	GSM2263040	Monocyte	Blood	H3K9me3	BG_d1_H3K9me3
72234	GSE85246	GSM2263039	Monocyte	Blood	H3K4me3	BG_d1_rep2_H3K4me3
72266	GSE85246	GSM2262994	Monocyte	Blood	H3K4me1	BG_d1_rep2_H3K4me1
72268	GSE85246	GSM2262991	Monocyte	Blood	H3K4me1	BG_d1_rep1_H3K4me1
72279	GSE85246	GSM2262975	Monocyte	Blood	H3K27me3	BG_d1_H3K27me3
65292	GSE87218	GSM2325680	Monocyte	Blood	ATAC-seq	ATAC_BG_4h_8450
66443	GSE85246	GSM2262944	Monocyte	Blood	H3K27ac	BG_4h_rep2_H3K27ac
66444	GSE85246	GSM2262938	Monocyte	Blood	H3K27ac	BG_4h_rep1_H3K27ac
72229	GSE85246	GSM2263045	Monocyte	Blood	H3K4me1	BG_4h_rep1_H3K4me1
72241	GSE85246	GSM2263028	Monocyte	Blood	H3K9me3	BG_4h_H3K9me3
72248	GSE85246	GSM2263017	Monocyte	Blood	H3K27me3	BG_4h_H3K27me3
72252	GSE85246	GSM2263013	Monocyte	Blood	H3K4me1	BG_4h_rep2_H3K4me1
72283	GSE85246	GSM2262971	Monocyte	Blood	H3K4me3	BG_4h_rep2_H3K4me3
72313	GSE85246	GSM2262927	Monocyte	Blood	H3K4me3	BG_4h_rep1_H3K4me3

Supplemental Table S3 (Continued). The accession numbers for histone modification ChIP-seq datasets used to evaluate ATAC-seq analysis in human monocytes.

State	Total	GC	H2az	H3K4me1	H3K4me3	H3K27ac	H3K27me3
1 background	0.373	0.355	0.229	0.239	0.212	0.182	0.320
2 nucleosome	0.588	0.415	0.529	0.562	0.504	0.476	0.639
3 center	0.039	0.464	0.241	0.199	0.284	0.342	0.041

State	H3K36me3	H3K4me2	H3K79me2	H3K9ac	H3K9me3	H4K20me1
1 background	0.347	0.178	0.294	0.163	0.359	0.314
2 nucleosome	0.584	0.490	0.557	0.455	0.579	0.627
3 center	0.069	0.332	0.149	0.381	0.062	0.059

State	ActivePromoters	Heterochrom	Insulator	PoisedPromoter	Repetitive	Repressed	StrongEnhancer
1 background	0.052	0.347	0.123	0.137	0.485	0.346	0.114
2 nucleosome	0.167	0.636	0.487	0.424	0.489	0.628	0.422
3 center	0.780	0.017	0.390	0.439	0.026	0.027	0.464

State	Txn_elongation	Txn_transition	WeakEnhancer	WeakPromoter	WeakTxn
1 background	0.417	0.294	0.218	0.149	0.398
2 nucleosome	0.579	0.657	0.590	0.463	0.590
3 center	0.005	0.050	0.192	0.388	0.012

Supplemental Table S4. Overlap between various chromatin features and HMMRATAC state annotations based on ATAC-Seq.
